# The Natural History of IgE-Mediated Food Allergy: Can Skin Prick Tests and Serum-Specific IgE Predict the Resolution of Food Allergy?

**DOI:** 10.3390/ijerph10105039

**Published:** 2013-10-15

**Authors:** Rachel L. Peters, Lyle C. Gurrin, Shyamali C. Dharmage, Jennifer J. Koplin, Katrina J. Allen

**Affiliations:** 1 Murdoch Childrens Research Institute, Parkville, VIC 3052, Australia; E-Mails: rachel.peters@mcri.edu.au (R.L.P.); lgurrin@unimelb.edu.au (L.C.G.); s.dharmage@unimelb.edu.au (S.C.D.); Jennifer.koplin@mcri.edu.au (J.J.K.); 2 Department of Paediatrics, University of Melbourne, Parkville, VIC 3052, Australia; 3 Centre for Molecular, Environmental, Genetic and Analytic Epidemiology, University of Melbourne, Parkville, VIC 3010, Australia; 4 Department of Allergy and Immunology, Royal Children’s Hospital, Parkville, VIC 3052, Australia

**Keywords:** food allergy, natural history, tolerance, skin prick test, serum-specific immunoglobulin E, hen’s egg, peanut, cow’s milk

## Abstract

IgE-mediated food allergy is a transient condition for some children, however there are few indices to predict when and in whom food allergy will resolve. Skin prick test (SPT) and serum-specific IgE levels (sIgE) are usually monitored in the management of food allergy and are used to predict the development of tolerance or persistence of food allergy. The aim of this article is to review the published literature that investigated the predictive value of SPT and sIgE in development of tolerance in children with a previous diagnosis of peanut, egg and milk allergy. A systematic search identified twenty-six studies, of which most reported SPT or sIgE thresholds which predicted persistent or resolved allergy. However, results were inconsistent between studies. Previous research was hampered by several limitations including the absence of gold standard test to diagnose food allergy or tolerance, biased samples in retrospective audits and lack of systematic protocols for triggering re-challenges. There is a need for population-based, prospective studies that use the gold standard oral food challenge (OFC) to diagnose food allergy at baseline and follow-up to develop SPT and sIgE thresholds that predict the course of food allergy.

## 1. Introduction

IgE-mediated food allergy is an increasing public health concern in the pediatric population. It commonly presents in infancy and together with eczema, are often the first manifestations of the atopic march, predisposing children to develop asthma and other allergic diseases later in life [1,2,3,4]. Although infants can, in theory, be allergic to any food, the majority of reactions are to one of nine foods: peanut, hen’s egg, cow’s milk, wheat, soya, tree nuts, sesame, shellfish and kiwi fruit [5]. Reports on the prevalence of food allergy vary by region and food, as well as study design and the measure of food allergy [6,7]. A recent systematic review reported that at least 1%–2% and up to 10% of the US population suffers from food allergies, although this estimate includes self-report, skin prick test (SPT), serum-specific IgE (sIgE) and oral food challenges (OFC) as measures of food allergy [7]. More recently, an Australian study found that 10% of 12-month old infants were challenge-confirmed allergic to one or more foods [8].

Food allergy is a transient condition for some children, although reports on the rate of resolution vary between studies. Around 20% of peanut allergy resolves in childhood [9,10,11,12], although some reports suggest that up to 50% of peanut allergy is transient [13,14,15]. Egg and milk allergy generally have a better prognosis with the majority of children expected to outgrow their allergy by school age [16,17,18,19,20,21]. Recent reports, however, suggest that for both egg and milk allergy, resolution is not occurring as quickly as previously thought, with allergy persisting well into adolescence and beyond [22,23]. It is not clear if the natural history of food allergy is changing or if it is simply a reflection of different study designs and populations. The latter studies were recruited from tertiary referral centers and may be overrepresented by children with more severe food allergy, skewing the rate of resolution.

At this stage, there are few indices to predict when and in whom tolerance will develop. This presents the problem of determining the optimal time to subject the child to further OFC while considering their existing risk of reaction. Delaying OFC could subject the child to further unnecessary dietary restrictions and reduced quality of life. Conversely frequent OFC is resource intensive and potentially exposing the child to unnecessary adverse reactions. Currently the only tool available for monitoring the natural history is IgE levels, either by SPT or sIgE. It is common practice for clinicians to monitor IgE levels every 1–2 years and if they drop below a certain threshold (*i.e*., the 95% PPV for diagnosis of food allergy) then a food challenge will be ordered to confirm or refute the presence of food allergy. Alternatively if SPT or sIgE to that particular food is negative, or there is a history of accidental exposure without reaction which supports liberation of the diet, then home introduction may be offered without the need for formal OFC. It should be noted that despite low SPT, that some children may not be suitable candidates for home introduction, as in the case of unstable asthma or past history of anaphylaxis. Finally, monitoring SPT and sIgE may help identify children who are unlikely to develop tolerance, and could therefore be considered for interventions such as oral immunotherapy (OIT).

SPT and sIgE thresholds with 95% positive predictive value (PPV) for food allergy are frequently utilized in clinical practice to reduce the need for OFC. Children with SPT or sIgE level above these thresholds have a high probability of OFC-confirmed food allergy at diagnosis [24,25,26,27,28,29,30]. In a similar fashion, SPT and sIgE are used for following the natural history of the condition to assess whether tolerance may have developed. If carefully and systematically measured 95% PPV thresholds were developed, they could be used to determine the optimal time to review children and the necessity of repeat OFC in food allergic children.

We have previously published a systematic review on SPT with 95% PPV to diagnose food allergy in children [31]. The aim of this paper is to review the current literature on the relationship between SPT and sIgE and 95% PPV of these measures for tolerance development of childhood IgE-mediated food allergy, *i.e*., in a child previously diagnosed with food allergy, what are the 95% PPV for persistent food allergy or the development of tolerance? This review focuses on peanut, egg and milk allergy as they are responsible for the majority of allergic reactions in childhood.

## 2. Publications that Reported on SPT, sIgE and the Development of Tolerance in Food Allergic Children

A search of PubMed, Medline and Embase was conducted using the search terms “food allergy” or “food hypersensitivity” and “immune tolerance” or “prognosis” or “natural history” and “immunoglobulin E” or “skin test” and “peanut” or “egg” or “milk”. Studies were included if they met the following criteria: (1) contained a sample of children diagnosed with IgE-mediated food allergy; (2) tested for persistent or resolved food allergy by OFC and (3) investigated the relationship between either the change in SPT and sIgE over the course of follow-up, or SPT and sIgE measured at follow-up, and whether it could predict persistent or resolved food allergy. Studies that included both children with IgE- and non-IgE mediated food allergy, yet didn’t differentiate between these two types of food allergy in the results, were excluded from the review. Studies that reported SPT wheal size as grades, rather than absolute numbers, were also excluded.

The search of the electronic databases produced 386 results. Three-hundred and twenty-seven were immediately excluded because they were oral immunotherapy trials or review articles, did not examine egg, peanut or milk allergy, or did not describe the association between SPT or sIgE and the prognosis of food allergy. The remaining 59 articles were retrieved. A further 40 articles were excluded from the review because they did not meet the inclusion criteria. Reference lists of included articles and review articles were manually searched for potentially relevant papers which yielded another seven articles that met the inclusion criteria. A total of 26 articles are included in this review. They were published between 1982 and 2013.

## 3. How Comparable are the Studies: Limitations of and Variations across the Studies

On reviewing the literature, several limitations of previous studies have become evident which hinders comparisons between studies. These limitations include the absence of the gold standard test to diagnose food allergy or tolerance, biased samples in retrospective audits and lack of systematic protocols for triggering re-challenges. Study designs were also heterogeneous with regards to subject selection, age of participants and period of follow-up, making comparisons between studies difficult.

Previous research suggests that if food allergy is to resolve then it is more likely to do so at a younger age [17,32,33]. If a study followed older children, they were less likely to observe the development of tolerance, compared to a study that had followed a cohort since infancy. The diagnostic cut offs of 95% PPV of SPT wheal size and sIgE are age-dependent, that is, thresholds differ when stratified by age (infants *versus* children and adolescents) [29,34,35]. Therefore it is likely that the prognostic cut offs of 95% PPV of SPT and sIgE may also vary with age. Both these factors suggest that studies examining the natural history of food allergy should be restricted to children of a similar age to ensure homogeneity in the development of PPVs, which was not usually the case with the studies considered in this review.

As with all food allergy research, the method to diagnose food allergy underpins the reliability of the results. The diagnosis of food allergy at baseline varied with only few studies having confirmed food allergy at baseline with the gold standard OFC. Many studies were retrospective and recruited children with a previous diagnosis of food allergy, based on clinical history and having a positive SPT or sIgE to the food, with or without OFC. Some studies included participants who had positive SPT or sIgE but no known history of exposure to the allergen, or participants who had SPT or sIgE greater than a particular threshold [10,22,36]. With each of these scenarios, there is a possibility that some children were only sensitised, but never actually allergic at baseline, which creates selection bias by including non-allergic participants.

In addition, retrospective audits may be subject to selection bias and inclusion criteria are often poorly defined. Children with complex allergic disease (e.g., multiple food allergies or coexisting atopic disease) are likely to be over-represented among studies that recruited participants from tertiary care settings compared to studies where participants were drawn from the general community. Resolution of milk allergy is reported to be higher in population-based studies, compared to those recruited from tertiary allergy clinics as they include the full spectrum of cases [17,33,37,38]. High loss to follow up may also bias the results, particularly in retrospective studies recruited from tertiary clinics, where children who have developed tolerance may be less likely to return for follow up assessment, and therefore the true rate of tolerance may be underestimated.

Although most studies confirmed the resolution of food allergy with OFC, there was a lack of systematic protocols recording reasons for triggering OFC or time intervals for repeat OFC for tolerance detection. Several studies stated that children were offered repeat OFC when clinically indicated, but did not always define what that meant [14,39,40]. In the case of retrospective studies, post hoc definition of reasons for challenge is likely to be problematic. Based on the current standard of practice, potential triggers for challenge could include SPT or sIgE approaching a negative result or if the child had an accidental exposure with no reaction. Other scenarios encountered in this review were to only offer OFC when SPT or sIgE fell below a predetermined threshold or assuming children with SPT or sIgE above a particular threshold were still allergic. Using a predetermined SPT or sIgE threshold as a marker for allergy or tolerance, in a study examining the usefulness of the test to predict that outcome, may bias the results as it acts as a self-fulfilling prophecy, e.g., the test will have high predictability to persistent allergy when all participants above that level are assumed to remain allergic. In addition, only offering OFC if SPT or sIgE is negative is likely to miss some cases of tolerance that have developed despite the test result remaining elevated.

## 4. What do These Studies Tell Us?

Twenty-six studies were identified that examined the use of SPT or sIgE at or during the course of follow-up and all except four studies reported that there was an association between these tests and the prognosis of food allergy [12,39,41,42]. At follow-up, SPT and sIgE was larger in children whose food allergy persisted [20,43,44,45,46] and SPT and sIgE decreased over time in children with resolved allergy [10,15,22,47,48]. sIgE was also observed to decrease in children with persistent food allergy, but this decrease was not as sharp as that observed in children with resolved food allergy [22,33].

### 4.1. Peanut Allergy

Eight studies examined the association between SPT and/or sIgE and the prognosis of peanut allergy. Six studies showed SPT or sIgE thresholds that predicted persistent or resolved peanut allergy whilst two studies found no association between SPT or sIgE and development of tolerance to peanut [12,39]. Characteristics and results of these studies are presented in Table 1.

Most studies were retrospective, that is, either retrospective chart reviews or recruited participants retrospectively with prospective follow-up. Only two studies used OFC to confirm the diagnosis of peanut allergy in all participants at baseline, but unfortunately these studies were both limited by very small sample sizes and neither were able to demonstrate an association between SPT or sIgE and resolution of peanut allergy [12,39]. Several studies used a predetermined SPT/sIgE threshold to either diagnose peanut allergy at baseline or persistent allergy at follow-up or simply excluded participants with sIgE above a particular threshold, which makes comparisons between these studies difficult [9,10,15]. At follow-up, only one study offered OFC to all participants irrespective of SPT wheal size, however parents were given the option to opt-out if SPT ≥ 8 mm [15]. The remaining studies only offered OFC if SPT or sIgE fell below a pre-determined threshold, which varied between studies [9,10,13,14,15,40].

In the study by Ho *et al*., 267 children under 2 years of age were recruited with a diagnosis of peanut allergy based on SPT ≥ 4 mm. Children were reviewed every 1–2 years, for up to 8 years, and offered OFC when SPT fell below the previously reported 95% PPV for their age. The authors reported that SPT ≥ 6 mm, sIgE ≥ 3 kU_A_/L before 2 years of age or an increase in peanut SPT of 3 mm or more between 1 and 4 years of age was predictive of persistent peanut allergy. Decreasing SPT was a good indicator of tolerance development but as the authors themselves discuss, those with a persistently elevated SPT were not routinely offered OFC, so the results were biased to persistence of allergic disease in those with persistently elevated SPT and overestimated the PPV [10].

**Table 1:** Studies reporting on SPT and sIgE and the prognosis of PEANUT allergy.

Author	N	Study design	Diagnosis of food allergy	Age ^1^	Follow-up period	Follow-up diagnosis	Resolution	SPT (mm)	sIgE (kU_A_/L)
Mudd *et al*. 2009 [39]	28	Retrospective chart review	OFC	5 years	2.8 years (range 0.7–10 years)	OFC if clinically indicated (time, exposure and symptom history since last OFC or sIgE)	46%		Not predictive of tolerance
Ho *et al*. 2008 [10]	267	Longitudinal	SPT ≥ 4 mm	<2 years	Reviewed every 1–2 years for up to 8 years.	OFC offered when SPT < 4 mm (<2 years old) or 8 mm (>2 years old)	18%	Increase SPT ≥ 3 between 1 and 4 years of age is predictor of persistence SPT ≥ 6 before 2 years predictive of persistent allergy	sIgE ≥ 3 before 2 years predictive of persistent allergy
Nolan *et al*. 2007 [15]	54	Prospective	Clinical history or positive SPT/sIgE with no history of ingestion. Excluded if sIgE >10 kU_A_/L, reaction in previous 2 years or previous severe reaction	6.3 years (range 3.7–14.8 years)	NR	OFC (irrespective of SPT however parents could opt-out if SPT ≥ 8 mm	53%	SPT ≥ 7 mm, 93% PPV	
Perry *et al*. 2004 [14]	173	Retrospective chart review	Clinical history or SPT/sIgE with no history of ingestion	4.8 years	NR	OFC (if clinically indicated or when sIgE 25% of previously reported 95% PPV)	59%		<2, 50% probability of tolerance
				(range 1–43 years)					
Fleischer *et al*. 2003 [13]	84	Retrospective chart review	Clinical history and SPT/sIgE or OFC; or no ingestion but positive SPT/sIgE	>4 years	NR	OFC or DBPCFC if sIgE < 5	55%		sIgE < 2 more likely to develop tolerance than sIgE 2–5 kU/L
Skolnick *et al*. 2001 [9]	223	Retrospective follow-up	Clinical history and positive SPT, sIgE or OFC; or positive SPT and sIgE but no ingestion	1.5 years (2 months–15 years)	NR	OFC. sIgE > 20 assumed allergic sIgE > 10 and severe reaction, no OFC (138 not challenged)	21.5%		sIgE ≥ 5 64% PPV and 61% NPV
Hourihane *et al*. 1998 [40]	30	Case-control	Clinical history	5 years (range 2–10 years)	NR	OFC if clinically indicated (recent non-eventful exposure or negative SPT)	50%	SPT ≥ 6 100% PPV and 80% NPV	Not predictive of tolerance
Sampson 1989 [12]	10	Retrospective	DBPCFC	7.9 years (range 3–18 years)	1–3 years	DBPCFC or accidental ingestion	20%	Not predictive of tolerance	

^1^ Age at diagnosis or entry into study; reported as mean or median (range); NR: not reported.

Nolan *et al*. completed a prospective study of children referred to a tertiary allergy clinic with a history of peanut allergy or positive sensitisation test with no history of ingestion. Children were excluded if they had a severe reaction within the preceding 2 years or if sIgE > 10 kU_A_/L. All children were offered OFC, however parents of children with SPT ≥ 8 mm were given the option to opt-out of the study considering the child’s high risk of reaction (n = 2). Therefore, the more severe cases of peanut allergy were not represented in this study, limiting the generalisability of results to these cases. In this study, 27 of 51 children were considered to have developed tolerance to peanut on OFC, however 16 of the tolerant children were only sensitised at entry into the study with no known exposure to peanut. It is possible that these children were never allergic to peanut. SPT ≥ 7 mm had 93% PPV to persistent peanut allergy although this is limited to children with sIgE < 10 kU_A_/L [15]. A similar result was reported from a case-control study of 30 children with persistent (n = 15) and resolved (n = 15) peanut allergy; SPT ≥ 6 mm had 100% PPV to persistent peanut allergy and 80% NPV to resolved peanut allergy [40].

A further three studies have reported sIgE thresholds that predict persistent or resolved peanut allergy, however the results are likely to be biased towards persistent allergy and overestimated the PPV, because OFC were only offered if sIgE was low. Skolnick *et al*. reported sIgE ≥ 5 kU_A_/L to have 64% PPV to persistent allergy in a retrospective follow-up of 233 children. However, over half of this sample (n = 138) were not eligible for OFC at follow-up on the basis of high sIgE [9]. In a retrospective chart review of 173 children diagnosed with peanut allergy by clinical history or positive SPT/sIgE with no history of ingestion, Perry *et al*. reported that children with sIgE < 2 kU_A_/L at follow-up should be considered for OFC because it was associated with a 50% probability of tolerance. However, participants were mostly offered OFC if sIgE was negative or fell to 25% of previously reported 95% PPVs. It is possible that they have underestimated the resolution of peanut allergy and in turn, the PPV, by limiting their challenge criteria to children with negative or low sIgE [14]. Fleischer *et al*. also reported, in a retrospective chart review of 84 children, that those with sIgE < 2 kU_A_/L were more likely to develop tolerance, however again OFC was only conducted if sIgE was below a given threshold (<5 kU_A_/L) [13].

These results suggest that SPT and sIgE may be useful in predicting the persistence of peanut allergy, however most existing studies are biased towards persistent allergy which is likely to overestimate the PPV, because children with high SPT or sIgE were assumed to have persistent allergy or were excluded. More research is needed to qualify if these tests are useful in identifying which children are likely to outgrow their peanut allergy. Furthermore, all studies recruited participants from tertiary allergy clinics, which are likely to be overrepresented by children from the more severe end of the spectrum of food allergy, thus limiting the generalizability of the results to community settings. There is currently no research examining the natural history of peanut allergy at the population-level, irrespective of SPT wheal size and sIgE level.

### 4.2. Egg Allergy

Eleven studies reported on the use of SPT and sIgE during the course of follow-up, and its association with persistent or resolved egg allergy (Table 2). Three studies did not find any significant association between SPT or sIgE and prognosis of egg allergy [12,39,41].

**Table 2:** Studies reporting on SPT and sIgE and the prognosis of EGG allergy.

Author	N	Study design	Diagnosis of food allergy	Age ^1^	Period of follow-up	Follow-up diagnosis	Tolerance	SPT (mm)	sIgE (kU_A_/L)
Montesinos *et al*. 2010 [48]	42	Retrospective follow-up	Clinical history and positive SPT/sIgE and OFC	15.7 months (range 8–27.5 months)	49 months (range 15–118.6 months)	OFC annually unless reaction in previous 3 months and positive SPT/sIgE	50%		≤2 years: 1.37, 96% PPV 0.36, 66% NPV
									2–3 years: 1.52, 100% PPV 0.54, 75% NPV
									3–4 years: 1.35, 100% PPV 0.36, 75% NPV
									4–5 years: 2.59, 100% PPV 0.96, 60% NPV
									>5 years: 1.84, 100% PPV 0.65, 100% NPV
Dieguez *et al*. 2009 [20]	157	Retrospective follow-up	Clinical history and positive SPT or sIgE, or OFC	2.5 years (range 1–16 years)	NR	DBPCFC	36%	≥7, 90% PPV, LR + 6.7	≥1.5 90% PPV, LR + 5.5
								≥9, 96% PPV, LR + 12.3	
Kim 2009 [41]	106	Retrospective	sIgE > 2	<2 years	Median 49 months (range 23–132 months)	Home challenge test if sIgE < 1.5 or accidental ingestion without reaction	58%		Peak sIgE not predictive of outcome
Mudd 2009 [39]	38	Retrospective chart review	OFC	5 years	2.8 years	OFC when clinically indicated	47%		Not predictive of tolerance
Savage *et al*. 2007 [22]	881	Retrospective chart review	Clinical history or sIgE > 2 and no known exposure	14 months (IQR 10, 23 months)	Median 59 months (range 5–285 months)	OFC if sIgE < 2 kU_A_/L	68%		Rate of tolerance inversely related to peak sIgE. sIgE > 50 marker for persistent allergy
Perry *et al*. 2004 [14]	138	Retrospective chart review	Clinical history or SPT/sIgE	NR	NR	OFC when clinically indicated or sIgE < 95% PPV	57%		sIgE < 2 50% probability of tolerance
Shek *et al*. 2004 [33]	88	Retrospective	DBPCFC	6 months–17 years	Up to 10 years	DBPCFC	32%		sIgE decrease by 50% in 12 months: 0.52 probability of tolerance
									sIgE decrease by 90% in 12 months: 0.78 probability of tolerance
Boyano-martinez *et al*. 2002 [49]	59	Prospective	OFC (n = 35) or recent reaction and positive sIgE	<2 years	Median 32 months	OFC every 1–3 years depending on previous symptoms	59%	SPT < 6 increased likelihood (HR) of tolerance by 3.74 (95% CI 1.60–8.74).	sIgE predicted tolerance only in children with cutaneous symptoms
Crespo *et al*. 1994 [43]	40		OFC	1 year	2.5 years	OFC	38%		sIgE ≥ 1.2 ku_A_/L 92% PPV
Sampson 1989 [12]	59	Retrospective	DBPCFC	7.9 years (range 3–18 years)	1–3 years	DBPCFC or accidental ingestion	24%	Not predictive of tolerance	
Ford 1982 [47]	25		DBPCFC	17 months (range 6 months–10 years)	2–2.5 years	OFC	44%	Decreased in resolved allergy	

NR: not reported; IQR: interquartile range; HR: hazard ratio; ^1^ Age at diagnosis or entry into study; reported as mean or median (range).

Boyano Martinez conducted the only prospective study on the natural history of egg allergy. Fifty-nine children under 2 years of age were recruited from a paediatric allergy clinic, with a previous diagnosis of egg allergy on the basis of OFC or meeting several criteria associated with a high probability of reaction (e.g., positive sIgE, recent reaction, previous anaphylaxis). Children were reviewed every 6–12 months depending on their age and offered OFC every 1–3 years depending on their previous reactions to egg. During follow-up, egg allergy resolved in 59%. SPT < 6 mm at follow-up increased the likelihood (hazard ratio (HR)) of tolerance by 3.74 (95% CI 1.60–8.74). Whilst it is useful to know that SPT below a particular level increases the odds of tolerance, it is less useful in a clinical setting without the accompanying sensitivity, specificity and positive and negative predictive values, which were not reported in this study. Magnitude of sIgE was also associated with tolerance, however only in children with cutaneous symptoms at the time of diagnosis. For every 0.1 unit decrease in sIgE, the likelihood (HR) of tolerance increased by 1.17 (95% CI 1.05–1.30). No such association was observed in children with respiratory or gastrointestinal symptoms on their index reaction to egg [49].

Thresholds of sIgE with high PPV to persistent egg allergy were reported in three studies and ranged from ≥1.2 to 2.59 kU_A_/L [20,43,48]. Montesinos *et al*. conducted a retrospective follow-up study of 42 children, (mean age 15.7 months, range 8–27.5) with a previous diagnosis of egg allergy confirmed by OFC and all children were rechallenged annually unless they had had a reaction within the previous 3 months. Although not explicitly stated, from the available data, it appears that OFC at follow-up was undertaken irrespective of sIgE. The authors reported thresholds with ≥95% PPV to persistent egg allergy for different periods of follow-up which ranged from <2 to >5 years. sIgE thresholds with 95% PPV ranged from ≥1.35–2.59 kU_A_/L. NPVs to predict resolved egg allergy were also reported, but only reached high probability (100% NPV) at ≥5 years of follow-up when sIgE < 0.65 kU_A_/L [48]. Crespo *et al*. reported a similar threshold of sIgE ≥ 1.2 kU_A_/L to have 92% PPV to persistent egg allergy after 2.5 years of follow-up [43].

Diguez *et al*. conducted a cross-sectional study on 157 children with a previous diagnosis of egg allergy on the basis of clinical history and positive SPT/sIgE, or OFC when necessary. Persistence or tolerance was determined by DBPCFC in all participants. SPT ≥ 7 mm and sIgE ≥ 1.5 kU_A_/L at follow-up had 90% PPV to persistent egg allergy. This is one of the few studies to also report the positive likelihood ratio which was 6.7 and 5.5 respectively, for the aforementioned SPT and sIgE thresholds [20]. PPVs are dependent upon the underlying prevalence of disease, unlike the likelihood ratio which is derived from the sensitivity and specificity. The likelihood ratio allows the clinician to transfer results from one setting to their own, and develop the probability of disease for each patient depending on their pre-test probability of disease [35]. These three studies were recruited from tertiary allergy clinics where between 36% and 50% of egg allergy resolved, and may not be applicable in settings where the prevalence of egg allergy is lower, or rate of resolution is higher, for example in a community setting.

Two studies reported on whether peak sIgE was associated with the development of tolerance to egg in egg allergic patients, but with differing results. Savage *et al*. conducted a large, retrospective chart review of 881 children with egg allergy, diagnosed by clinical history or sIgE > 2 kU_A_/L with no known tolerance to egg. As for Ho *et al*.’s peanut allergy study [10], assessment of tolerance to egg by OFC was only activated when the IgE fell below a certain threshold (*i.e*., when sIgE < 2 kU_A_/L) or following uneventful accidental exposure. The authors found that sIgE decreased in both children with persistent and resolved egg allergy, however the rate of decrease was sharper in those with resolved allergy. Children with persistent egg allergy had larger sIgE levels in the first two years of life compared to children with transient allergy and this difference was maintained through to 18 years. Peak sIgE was inversely related to the probability of tolerance, *i.e*., the higher the peak sIgE, the less likely that the child would outgrow egg allergy. Children with peak sIgE < 2 kU_A_/L had the fastest rates of tolerance development. Although this study is very important because of the large sample size, it is limited by biasing towards persistent allergic disease as participants were generally offered OFC when sIgE fell below 2 kU_A_/L [22]. In contrast, in a smaller retrospective study of 106 children with egg allergy defined as sIgE ≥ 2 kU_A_/L, peak sIgE was not associated with persistent allergy. A limitation of this study was that egg allergy or tolerance was not confirmed by hospital-based OFC at diagnosis or follow-up. Tolerance was confirmed by home challenge test only when sIgE < 1.5 kU_A_/L [41].

Two studies reported SPT or sIgE levels at follow-up that were associated with a child’s probability of having developed tolerance to egg. The study by Perry *et al*. (previously described for peanut allergy) reported that sIgE < 2 kU_A_/L was associated with a 50% probability of tolerance [22]. Shek *et al*. reported results from a retrospective study of 88 children and found that the degree of change in decrease in sIgE was associated with tolerance in children who were under 4 years of age at the time of their first OFC, but the association was weaker in children over 4 years of age. In children less than 4 years of age, a 50% decrease in sIgE in any 12 month period was associated with 52% probability of tolerance, whilst a 90% decrease was associated with a 78% probability of tolerance [33].

There is increasing evidence to suggest that 60%–80% of children allergic to raw egg are able to tolerate egg in baked foods, and furthermore, tolerance to baked egg is predictive of the resolution of egg allergy [50,51,52]. Improvement in the performance of 95% PPVs to the resolution of egg allergy may be achieved by stratifying upon egg allergy phenotype, *i.e*., raw and baked egg allergic *versus* raw egg allergic, baked egg tolerant.

Several studies reported on the use of SPT and sIgE to predict the persistence or resolution of egg allergy. Three studies supported the use of sIgE between 1.2 to 2.59 kU_A_/L at follow-up as a marker for persistent egg allergy [20,43,48], whilst others suggested that low or decreasing sIgE was predictive of resolved egg allergy. Only one study was prospective, and no studies were recruited from a population-based setting. There is a need for prospective, population-based studies using the gold standard OFC in all participants, irrespective of SPT and sIgE, to examine the predictive value of these tests to the prognosis of egg allergy.

### 4.3. Milk Allergy

Thirteen studies reported on the use of SPT and sIgE during follow-up, and its association with persistent or resolved milk allergy (Table 3). Two studies did not find any significant association between SPT or sIgE and prognosis of milk allergy; these studies both had small sample sizes (<30 participants) [12,42].

**Table 3:** Studies reporting on sIgE as a predictor of the prognosis of MILK allergy.

Author	N	Study design	Diagnosis	Age ^1^	Period of follow-up	Follow-up diagnosis	Tolerance	SPT (mm)	sIgE (kU_A_/L)
Yavuz 2013 [53]	148	Retrospective	Clinical history, positive SPT/sIgE and either positive OFC or history of anaphylaxis	1.6 years (IQR 0.9–3.1 years)	3.5 years (IQR 1.8–5.2 years)	OFC if sIgE low and no recent reaction	45%		Age-specific 95% PPVs to persistent allergy
									<1 years: 1.4
									<2 years: 9.3
									<4 years: 9.4
									<6 years:11
									All: 11
Suh 2011 [36]	115	Retrospective	Clinical history and sIgE > 0.35; or sIgE > 5	<24 months	Mean 47 months (range 24–114 months)	OFC or home introduction	41%		Higher peak sIgE in first 24 months reduced probability of tolerance.
Savilahti 2010 [46]	83	Prospective birth cohort (n = 6,209)	Open OFC and SPT ≥ 3 mm or sIgE ≥ 0.7	7 months	Up to 8 years	OFC or home introduction	78%		sIgE higher in those with persistent allergy
Santos 2010 [54]	66	Retrospective	OFC and SPT or sIgE	NR	Median 7 years	OFC if SPT or sIgE decreased and no recent reaction	50%	Peak > 10 mm reduced the likelihood of tolerance (HR 0.38, 95% CI 0.14–0.99)	Higher maximum sIgE over time reduced the likelihood of tolerance
Martorell 2008 [16]	170	Prospective	Clinical history or OFC, and SPT or sIgE	5.4 months (1–12 months)	Up to 4 years of age	OFC if no reaction in previous 3 months	82%		Age-specific 95% PPVs to persistent allergy
									12 months: 5.86
									18 months: 9.79
									24 months: 25.7
									36 months: 7.38
									48 months: 5.0
Skripak 2007 [23]	807	Retrospective review	Clinical history plus OFC or elimination diet	13 months (range 1–209 months)	Median 54 months	OFC if sIgE < 2; or home introduction	15%		Higher peak sIgE reduced probability of tolerance.
Saarinen 2005 [17]	86	Prospective birth cohort	Elimination challenge test	7 months	Up to 8 years	Home challenge test or OFC	85%	SPT 12 months after diagnosis: ≥7 mm AUC 0.84	
Perry 2004 [14]	166	Retrospective chart review	Clinical history or SPT/sIgE	NR	NR	OFC if clinically indicated	45%		<2 50% probability of tolerance
Garcia-Ara 2004 [44]	66	Prospective	OFC	4.8 months (range 1–11 months)	Mean 32.9 months (range 9–99 months)	OFC	68%		Age-specific 95% PPVsto persistent allergy
									13–18months: 2.7
									19–24months: 9
									26–36months: 24
Shek 2004 [33]	49	Retrospective	DBPCFC	34 months	Up to 10 years	DBPCFC	33%		sIgE decrease of 50% in 12 months—0.31 probability of tolerance
									sIgE decrease of 90% in 12months—0.66 probability of tolerance
Hill 1993 [45]	69	Prospective	OFC or SPT	20 months	Median 24 months	Home introduction or OFC at 5 years of age	22%		Lower in transient allergy
James 1992 [42]	29		DBPCFC	7 years (range 3–14 years)	Median 3 years	DBPCFC	38%	Not predictive of tolerance	Not predictive of tolerance
Sampson 1989 [12]	21	Retrospective	DBPCFC	7.9 years (range 3–18 years)	1–3 years	DBPCFC or accidental ingestion	19%	Not predictive of tolerance	

NR: not reported; HR: hazards ratio; ^1^ Age at diagnosis or entry into study; reported as mean or median (range).

There were three studies that reported milk-sIgE thresholds with 95% PPV to persistent milk allergy which stratified the 95% PPVs by age of the children during follow-up. Yavuz *et al*. conducted a retrospective study of 148 children diagnosed with milk allergy on the basis of clinical history, positive SPT or sIgE and either a positive OFC or history of anaphylaxis. Children were offered an OFC to test for tolerance when it was considered they had a 50% probability of tolerance, based on lack of recent reactions or low sIgE levels. sIgE thresholds with 95% PPV to persistent milk allergy were 1.4 kU_A_/L, 9.3 kU_A_/L, 9.4 kU_A_/L and 11.0 kU_A_/L for children <1, <2, <4, and <6 years of age respectively. Specificity was generally high at these thresholds, 89%–96%, but sensitivity was modest, 44%–90% [53].

In a prospective study by Martorell *et al*., 170 infants under 12 months diagnosed with milk allergy, on the basis of clinical history or OFC, were offered repeat OFCs periodically over 4 years, with the exception of those who experienced a reaction in the preceding 3 months. The authors reported thresholds with 95% PPV to persistent milk allergy for different ages between 12 and 48 months, which differed substantially by age. Thresholds with 95% PPV to persistent milk allergy were 5.86 kU_A_/L, 25.7 kU_A_/L, 7.38 kU_A_/L and 5.0 kU_A_/L at 12, 24, 36 and 48 months respectively [16].

Another study also reported that 95% PPVs to milk allergy persistence varied by age. In this study 66 infants aged under 12 months were recruited and milk allergy was confirmed by OFC in all participants at diagnosis and review, with the exception of those who had a reaction in the preceding 3 months. sIgE thresholds with 95% PPV to persistent milk allergy were 2.7 kU_A_/L, 9.0 kU_A_/L and 24.0 kU_A_/L at 13–18, 19–24 and 25–36 months respectively [44]. This supports the argument for the necessity of age-specific PPVs to predict the course of food allergy, however, even when restricted to similar ages of children, the 95% PPVs differ substantially between studies. This issue is common among studies reporting 95% PPVs at the diagnosis of food allergy and is largely due to differences in populations, study designs and prevalence of disease [31].

Several studies recommended optimal SPT or sIgE cut-offs from ROC analysis or thresholds with a hazard or odds ratio that indicate it was predictive of the outcome [14,17,36,54]. Often however, the sensitivity and specificity at the threshold was not reported, so although they may be useful to guide clinician’s decision making as to the likelihood of a reaction, these thresholds should not be used as a proxy for OFC as the number of false positives and negatives is not specified. Three studies reported that the higher the peak sIgE, the lower one’s probability of developing tolerance [23,36,54] and one study reported the same association with SPT [54]. Shek *et al*. reported that a 50% or 90% decrease in sIgE within a 12-month period was associated with a probability of achieving tolerance by 0.31 and 0.66, respectively [33].

There was one recently published study that did not meet the inclusion criteria because it examined SPT and sIgE at diagnosis, rather than at follow-up, to predict the course of milk allergy, but it is worth noting here. Wood *et al*. published results from an observational study of 293 children with milk allergy who were aged between 3 and 15 months at enrolment. The majority of children were followed-up until at least 4 years of age where tolerance was confirmed by either OFC or successful home introduction. Using proportional hazards regression, the authors found that baseline SPT, sIgE and severity of atopic dermatitis was predictive of resolved milk allergy. Although no specific diagnostic threshold was reported, the authors developed an online calculator that produced a graph of probability of resolution of milk allergy over time, based on SPT, sIgE and eczema at diagnosis [37]. This is a useful tool to individualize patient counseling and could be used to address parent questions relating to the probability and timing of resolution. It would be useful to replicate this for other foods. Other research teams have developed calculators for the diagnosis of food allergy using several routinely available clinical factors, including SPT, sIgE, symptoms, sex and age. Similar tools may be useful for predicting the development of tolerance [55].

Two studies were derived from birth cohorts and represent population-derived data with the full spectrum of disease, rather than only the severe end of the spectrum that are likely to dominate tertiary clinic samples [17,46]. These studies reported higher rates of resolution, but unfortunately did not present sufficient information on the predictive value of SPT or sIgE to resolution of milk allergy.

## 5. Summary and Future Directions

It is not currently clear why some children outgrow their food allergy and others do not. OFCs remain the gold standard for diagnosis of food allergy, as well as determining tolerance, yet are resource consuming and can result in severe reactions. Therefore, reliable criteria as to when to conduct a repeat OFC for tolerance detection or defer because of a high likelihood of persistent allergy are needed in clinical practice. Whilst there is modest evidence that SPT and sIgE may predict which children have a high probability of persistent food allergy, there is not a sufficient body of evidence to support the use of a particular threshold as a marker of resolved or persistent allergy and to avoid unnecessary OFC. Low or decreasing SPT and sIgE was associated with the development of tolerance, however there is little data to inform whether persistently elevated SPT or sIgE really predicts the persistence of allergy, since many studies assumed that those with elevated results were still allergic. The limitation of using sIgE for assessment of resolution of food allergy is the fact that to date no study has systematically assessed whether 95% PPV developed for food allergy diagnosis are also appropriate for assessment of resolution. The only way to systematically assess resolution would be to regularly challenge consecutive children irrespective of SPT or sIgE. Currently challenges are usually only considered when SPT/sIgE decrease in size or become negative and therefore are biased to the persistence of food allergy. Since most studies assessing SPT/sIgE for resolution undertake retrospective analysis of databases and do not provide the predetermined clinical criteria for ordering tolerance challenges the literature in this area is severely deficient.

This review has highlighted several limitations of previous research, which unfortunately may limit our ability to make any clinical recommendations. These include the absence of systematic application of the gold standard test, (OFC) to diagnose food allergy or tolerance irrespective of wheal size in order to generate non-biased estimates, biased participation in retrospective audits and lack of systematic protocols for triggering re-challenges. Future studies should ensure that they have taken appropriate measures to overcome these. Importantly to date, very few studies have undertaken follow-up OFC irrespective of SPT wheal size. Therefore, currently available data is likely to be significantly biased to the persistence of allergic disease and overestimate the PPV. This significant gap in the literature is important not just from a clinical prognostic perspective with regards to what we tell our patients, but also informs predicted outcomes for OFC which may be incorrectly assuming lower rates of resolution. Oral immunotherapy trials, which do undertake follow-up OFC irrespective of wheal size, are likely to offer important natural history information in control arms. Currently there are limited numbers of participants in placebo arms of RCTs, yet this data could be used for the systematic development of PPVs for food allergy resolution [56,57]. Data from these studies and in progress cohort studies such as the HealthNuts study which is capturing follow-up OFC data in all sensitised children at age 4 (with food allergy status initially determined by OFC at 12 months for all children) irrespective of SPT wheal size will provide a source of invaluable prognostic data [58].

There are several potential improvements for the prediction of tolerance that are yet to be sufficiently investigated. Whilst it is established that there are certain risk factors for the development of food allergy, for example eczema and family history, it is not yet clear whether the presence or absence of these risk factors are associated with the persistence or resolution of food allergy. Although several studies have examined these, results are largely inconsistent [22,41,49,54,59,60]. However, if these factors are demonstrated to offer predictive value to the course of food allergy, the sensitivity and specificity of SPT and sIgE may be further improved by stratifying upon these factors. Likewise, stratifying upon immunological factors that predict the risk of food allergy, e.g., serum cytokine profiles, may improve the predictive value of SPT and sIgE [61].

Component resolved diagnostics (e.g., sIgE to sub-fractions of major food allergens, for example Ara H 2 in peanuts), have been shown to improve the diagnosis of food allergy because they have higher sensitivity and specificity than peanut-sIgE alone [62]. Likewise they may play a significant role in predicting the likelihood of persistent or resolved peanut allergy. Individual cow’s milk allergens, particularly casein and lactalbumin, were shown to be good prognostic markers to persistent cow’s milk allergy, however their efficiency was comparable to milk-sIgE and at this stage, they are not recommend for daily practice due to increased laboratory costs with little additional benefit [44,63]. With regards to egg allergy, ovomucoid, which is one of the major allergens, may also be useful in the identification of children with persistent egg allergy. Children with persistent egg allergy tended to have higher concentrations of IgE antibodies to ovomucoid compared to children with transient egg allergy [64,65].

Another *in vitro* test that is being explored for the prediction of food allergy is the basophil activation test (BAT). Studies on the utility of BAT to predict food allergy and the development of tolerance are limited in number in comparison to SPT and sIgE. Initial studies have demonstrated moderate to high sensitivity and specificity of BAT to both the diagnosis of food allergy, and in particular, the resolution of milk allergy [66,67,68]. At this stage, BAT may be useful as a complement to conventional SPT and sIgE tests, and warrants further research.

Researchers are identifying new information about the genetic predisposition to food allergy and it is possible that genetics or gene-environment interactions may also play a role in the development of tolerance [69]. In the future, genotyping may assist in identifying children more likely to develop tolerance. In the study by Yavuz *et al*. (previously described), they found that the development of tolerance to cow’s milk was significantly higher in children with GG genotype at certain SNPs on STAT6, compared to AA + AG genotype, although there was no difference between their initial and peak sIgE levels between these two groups [53]. Nevertheless, this research is amongst the first to attempt to identify genetic predictors of tolerance development, of which there may be multiple. When used in conjunction with SPT, sIgE and possibly component resolved diagnostics, genetic risk stratification may improve the management of childhood food allergy.

## 6. Conclusions

In this study, we reviewed papers that examined the association between SPT or sIgE and the resolution of food allergy. Despite methodological and population differences, collectively these studies confirm that food allergy is a transient condition for some children and that SPT and sIgE evolve with the progression of the disease. Most studies reported thresholds that increased a patient’s probability of tolerance but the data on persistence is biased. Previous research is hampered by absence of OFC to diagnose food allergy or tolerance, highly-selective clinic samples, biased participation in retrospective studies, and lack of systematic protocols for triggering re-challenges There is a need for population-based food allergy studies, using OFC irrespective of SPT and sIgE to confirm all cases of food allergy at baseline and follow-up, to examine the ability of SPT and sIgE to predict the course of food allergy. This information will assist the allergist to determine optimal periods of follow-up, facilitate the decision of when to conduct future OFC to test for tolerance and avoid unnecessary OFC.
